# Capturing heterogeneity in PDX models: representation matters

**DOI:** 10.1038/s41467-024-47607-8

**Published:** 2024-05-31

**Authors:** Hari Shankar Sunil, Kathryn A. O’Donnell

**Affiliations:** 1 Department of Molecular Biology, UT Southwestern Medical Center, Dallas, TX USA; 2 Harold C. Simmons Comprehensive Cancer Center, UT Southwestern Medical Center, Dallas, TX USA; 3 Hamon Center for Regenerative Science and Medicine, UT Southwestern Medical Center, Dallas, TX USA

**Keywords:** Cancer models, Oncology

## Abstract

Patient derived tumor xenografts (PDXs) are important models for pre-clinical testing in cancer research and personalized medicine. PDXs often represent patient tumors with high similarity in terms of histology and driver mutations. However, certain limitations exist that warrant a detailed understanding of PDX heterogeneity and evolution. Hynds et al. demonstrate the relevance of primary tumor heterogeneity in PDX model establishment and explore multi-region sampling to determine the extent to which PDXs represent primary tumors.

Patient derived xenografts (PDXs) are thought to recapitulate the histology and genetic characteristics of patient tumors. They mimic tumor biology more closely than cell lines with respect to their in vivo cellular interactions. They also show similar drug responses as compared with patients, making them valuable models for pre-clinical drug testing and “avatars” for personalized medicine approaches^1–8^. However, PDX models do have limitations. For example, PDXs do not fully recapitulate all aspects of tumorigenesis, such as contributions from the immune system. Genomic evolution that occurs during the passage of PDXs in immunocompromised NOD/SCID IL2Rγ^null^ (NSG) mice results from de novo mutations and/or copy number alterations. However, there are conflicting reports in the literature regarding the degree of genomic evolution in PDX models^9–13^. It is, therefore, important to understand the genetic fidelity of PDXs to determine the extent to which these models truly represent primary tumors and their metastases.

Hynds and colleagues address three unresolved issues in the use of non-small cell lung cancer (NSCLC) PDX models including the extent of genomic bottlenecking during PDX establishment, the reproducibility of PDX derivation across spatially distinct regions of a tumor, and the emergence of genetic alterations during passaging of PDX models in mice^14^. Starting with 145 specimens from 44 patients undergoing surgical resection, the authors established 48 PDX models from 22 NSCLC patients enrolled in the TRACERx study, a large-scale research initiative that characterizes the evolutionary dynamic of NSCLC through a multi-region whole-exome sequencing (WES) approach^15^. They analyzed PDXs that were passaged in NSG mice for up to three passages from multiple regions of the primary tumors (Fig. 1). This was especially advantageous to assess changes in the PDXs due to expansion and engraftment from spatially distinct regions within the same patient tumor. Through these studies, the authors establish the importance of generating PDXs from multiple regions of primary lung tumors, which may capture the heterogeneities present to a greater extent, and which may be critical for the success of therapy resistance studies and personalized medicine approaches.

**Fig. 1:**
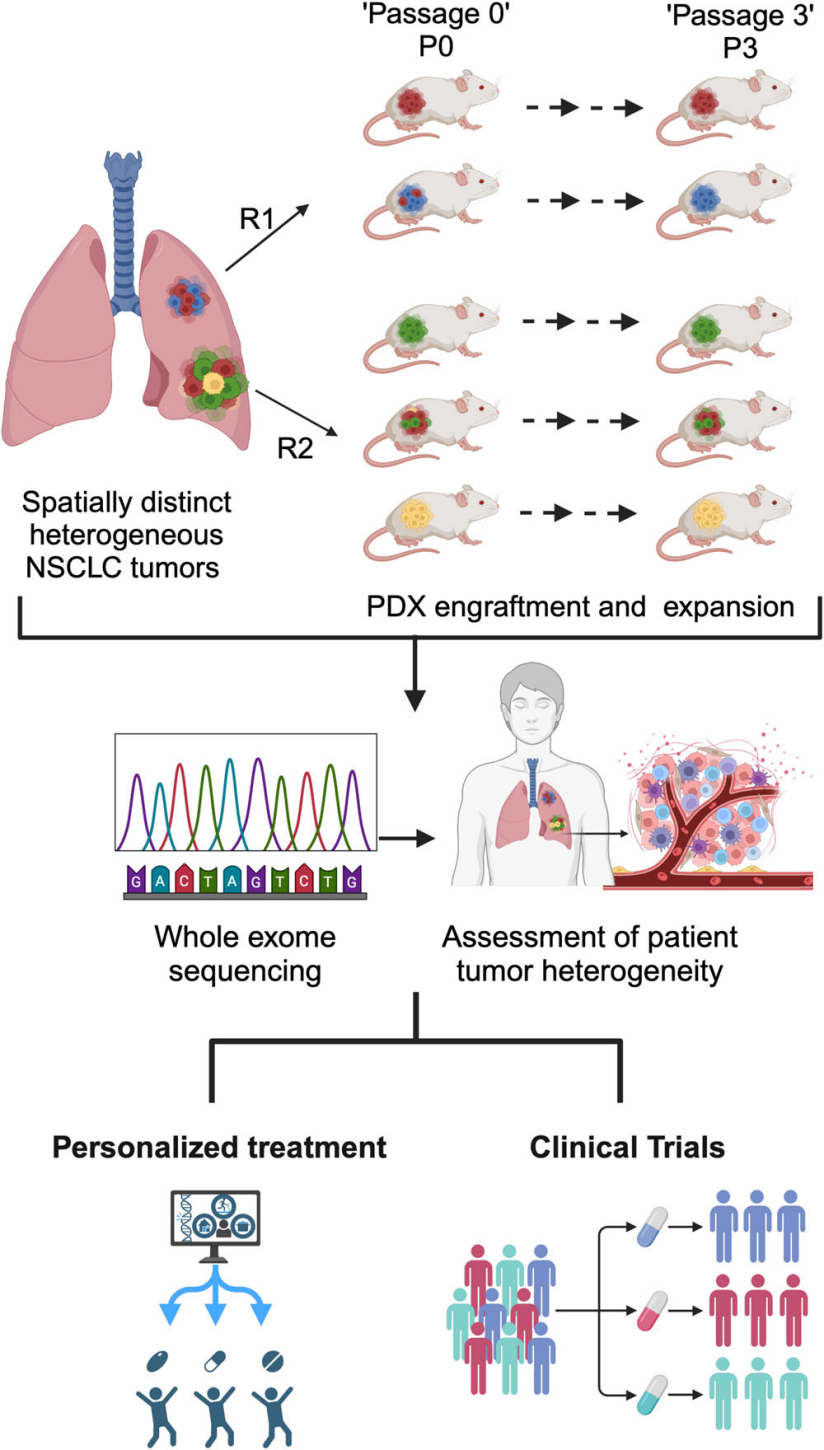
Patient derived xenografts are important for pre-clinical testing and for developing precision medicine strategies in lung cancer. In this study, Hynds et al. passage PDXs (P0-P3) through NSG mice from different regions of patient tumors (R1, R2, R3). By comparing whole exome sequencing (WES) data from initial passage zero (P0) through passage three (P3) PDX models and matched primary tumors from multiple tumor regions from the TRACERx study, the authors investigate several unresolved issues in the use of PDX models. Their findings reveal a genomic bottleneck upon engraftment as the major source of genomic variability between PDX models and their associated tumor region. Heterogeneous primary tumor regions often generated monoclonal PDX models. These data suggest that PDXs established from multiple tumor regions may more accurately recapitulate patient heterogeneity, and thereby provide important models for personalized medicine approaches. Fig. 1 was created with BioRender.com.

The authors provide a detailed analysis of engraftment characteristics of PDX-forming tumors with data thoughtfully presented by subtype, including lung adenocarcinoma (LUAD), lung squamous cell carcinoma (LUSC), and ‘other’ NSCLC histologies. They observed a strong correlation for the successful engraftment of PDXs with several factors, including disease-free survival, lesion size, copy number instability, loss of heterozygosity (LOH), *TP53* mutations, tumor purity, and T-cell infiltration. This analysis revealed previously unappreciated differences between the histological subtypes in multiple analyses. Their data also suggest that the PDX engraftment does not easily predict primary tumor clones with metastatic potential.

One aspect of this study that is certain to benefit all investigators who utilize PDX models is the development of an adapted mouse reference genome. To this end, the investigators performed whole exome sequencing (WES) on the NSG strain to inform the development of an NSG-adapted reference genome. They then employed filtration strategies that improved the removal of contaminating mouse reads from their sequencing studies. These efforts highlight a critical need for deriving a complete NSG reference genome assembly for use in PDX studies.

Importantly, the authors observed that a significant fraction of heterogeneous primary tumors gave rise to monoclonal PDXs ( > 60%) while the others gave rise to polyclonal PDXs, owing to the distinct subclones within the regions of origin in the patient tumors. This suggests a bottleneck event during PDX engraftment. A well-executed and thorough analysis of mutational distance and copy number distance revealed that the PDXs were more similar to their regions of origin as compared to regions of the tumor other than the one used to derive the PDX models (‘non-regions of origin’). This suggested that the patient tumors are spatially heterogeneous. Polyclonal PDXs were also more similar to their regions of origin than the monoclonal PDXs, thereby establishing the presence of multiple subclones within the same patient tumor. Interestingly, individual monoclonal PDXs were able to capture the major subclones as well as the minor subclones within the patient tumors, suggesting that multiple monoclonal PDXs would ideally be able to recapitulate the complex heterogeneities of patient tumors. Thus, the analysis of multiple monoclonal PDXs would reveal minor clones with distinct mutations and may potentially enable a more effective prediction of clinical outcomes. Moreover, this may assist in classifying patient tumors into specific treatment groups based on their unique mutational signatures that would have otherwise remained hidden within the patient tumors.

Hynds et al. further demonstrate that selective pressure during initial PDX engraftment and genomic evolution during the passaging through NSG mice determine the genomic characteristics of the PDXs. However, the accumulation of mutations during expansion in mice contributed less to the overall genomic distance of PDX models from primary tumors compared to the initial bottlenecking events. Although this indicates that the PDXs are less heterogenous than the patient tumors, the authors acknowledge that they did not analyze PDXs after passage 3 (P3), which may explain the differences seen in other studies that utilize later passage models.

In summary, the authors establish that distinct spatial regions of the same tumor can have divergent outcomes in PDX models, demonstrating the importance of generating PDXs from multiple regions of primary lung tumors. By carefully tracking mutations and copy number changes through engraftment and expansion in mice, this study reveals a genomic bottleneck that occurs during engraftment. The authors further suggest that PDX models lacking the full complement of subclones that were present in the primary tumor may present a challenge for personalized approaches where clonal selection in the PDX is thought to represent clonal selection in the patient. Thus, representation matters because it may affect therapy response. Given the underrepresentation of subclonal heterogeneity, this advocates for caution when interpreting results from single-region PDX models. Genomic changes acquired during both the establishment and passaging of PDXs should be evaluated.

Several important questions remain regarding NSCLC PDX models and represent a priority for future studies. For example, in addition to mutation and copy number, other multi-omics analyses of patients and PDX models and the association of ongoing genomic evolution in PDXs with drug response should be investigated. Taken together, PDX models will continue to be a mainstay in pre-clinical studies and cancer biology research for NSCLC and other tumor types. Experimental approaches that address the functional consequences of PDX monoclonality for therapy response and resistance are urgently needed and will ultimately advance the utility of these models.
